# Clinical–Radiomics Nomogram Based on ^18^F-FDG PET/CT for Distinguishing Lymphoma from Cancer of Unknown Primary in Head and Neck Metastatic Poorly Differentiated Cancer

**DOI:** 10.3390/bioengineering13101127

**Published:** 2026-09-27

**Authors:** Wen Chen, Mingzhen Xu, Bingxin Gu, Jianping Zhang, Xiaoqiang Shi, Ni Zhang, Shaoli Song

**Affiliations:** 1 Department of Nuclear Medicine, Fudan University Shanghai Cancer Center, Fudan University, Shanghai 200032, China; 2 College of Biomedical Engineering, Yiwu Research Institute, Fudan University, Shanghai 200433, China; 3 Department of Oncology, Shanghai Medical College, Fudan University, Shanghai 200032, China; 4 Center for Biomedical Imaging, Fudan University, Shanghai 200032, China; 5 Shanghai Engineering Research Center of Molecular Imaging Probes, Shanghai 200032, China; 6 Key Laboratory of Nuclear Physics and Ion-Beam Application (MOE), Fudan University, Shanghai 200433, China; 7 Department of Nuclear Medicine, Huadong Hospital Affiliated to Fudan University, Shanghai 200040, China

**Keywords:** PET/CT, nomogram, radiomics, cancer of unknown primary, lymphoma, heterogeneity index, machine learning

## Abstract

Objective: To develop and validate a clinical–radiomics nomogram based on ^18^F-FDG PET/CT for differentiating lymphoma from cancer of unknown primary (CUP) in head and neck metastatic poorly differentiated cancer. Methods: This retrospective study enrolled 192 patients, including 151 CUP and 41 lymphoma cases. Patients were randomly divided into a training (*n* = 134, 70%) and test cohorts (*n* = 58, 30%) using a fixed random seed for partitioning. Radiomic features were separately extracted from PET/CT images, and feature selection was performed using the least absolute shrinkage and selection operator algorithm. The optimal radiomics model was constructed via ten-fold cross-validation and comparative modeling with multiple machine learning methods. The clinical–radiomics nomogram was established by combining the radiomics model score and the clinical model score derived from gender and the metabolic heterogeneity index. The class distribution was preserved to reflect real-world clinical prevalence, consistent with recommendations for clinical prediction models. Results: The lymphoma group had a significantly higher female proportion (53.7%) than the CUP group (17.9%). The radiomics model yielded areas under the curve (AUC) of 0.916 in the training cohort and 0.812 in the test cohort. The final clinical–radiomics nomogram achieved higher and more robust performance, with AUCs of 0.924 (95% CI: 0.868–0.980) in the training set and 0.912 (95% CI: 0.837–0.987) in the test set, respectively. The standalone radiomics model exhibited a drop in sensitivity when evaluated in the test cohort, which directly demonstrates the incremental predictive value of integrating well-selected clinical parameters into the final model. Conclusions: The clinical–radiomics nomogram shows promising potential for differentiating lymphoma from CUP in poorly differentiated cervical lymph node malignancies. As a preliminary, single-center study, these findings warrant further validation in larger, multi-center cohorts before clinical implementation.

## 1. Introduction

Patients presenting with metastatic poorly differentiated malignancies of the head and neck pose a formidable diagnostic challenge. Standard clinical workup typically includes tumor biomarker testing, cross-sectional imaging, and pathological biopsy to identify the primary tumor site [1]. However, two distinct entities, lymphoma and head and neck cancer of unknown primary (HNCUP), are notoriously difficult to distinguish using conventional diagnostic approaches.

Accurate differentiation between these two entities is clinically imperative, as misclassification carries profound and potentially life-altering consequences. Fine-needle aspiration biopsy, the first-line diagnostic procedure for head and neck masses, frequently yields insufficient tissue for definitive histopathological and immunohistochemical characterization [2,3,4]. Compounded by overlapping imaging features between lymphoma and HNCUP, diagnostic uncertainty and misclassification are pervasive in clinical practice. When lymphoma is misdiagnosed as HNCUP, patients receive empiric platinum-based chemotherapy regimens that are ineffective for lymphoma and expose them to unnecessary toxicities [5,6]. Conversely, misclassifying HNCUP as lymphoma results in inappropriate chemotherapy administration while delaying the critical search for a primary tumor, which directly compromises long-term survival outcomes [7,8,9]. Thus, early and accurate distinction between lymphoma and HNCUP is a prerequisite for timely and personalized treatment decisions.

Current clinical practice for differentiating lymphoma from HNCUP follows a multistep diagnostic algorithm comprising serum tumor markers such as soluble interleukin-2 receptor (sIL-2R) and lactate dehydrogenase (LDH), human papilloma virus (HPV)/Epstein–Barr virus (EBV) serology and in situ hybridization, immunohistochemical analysis of biopsy specimens, and cross-sectional imaging. However, these conventional approaches frequently yield inconclusive results, particularly in patients with poorly differentiated malignancies. Tumor markers are inherently non-specific, HPV/EBV status may be negative in both conditions, and fine-needle aspiration specimens are often inadequate for definitive immunohistochemical subtyping [10,11]. Poorly differentiated tumors represent the most diagnostically challenging subset, as both lymphoma and HNCUP can exhibit indistinguishable morphological features, rendering standard pathological evaluation unreliable. This creates a critical diagnostic gray zone where conventional workup fails to establish a definitive diagnosis, highlighting the urgent need for additional non-invasive tools to aid clinical decision-making.

Radiomics, which involves the extraction of high-throughput quantitative features from medical images, can capture subtle tumor characteristics that are not perceptible to the human eye [12,13,14]. ^18^F-fluorodeoxyglucose positron emission tomography/computed tomography (^18^F-FDG PET/CT) is a powerful imaging modality in oncology that provides both anatomical detail and functional assessment of glucose metabolism, enabling non-invasive evaluation of intratumoral metabolic heterogeneity [15,16]. The diagnostic potential of PET/CT radiomics has been demonstrated in various lymphoma-related diagnostic tasks. Montes de Jesus et al. showed that radiomic features could distinguish follicular lymphoma from diffuse large B-cell lymphoma (DLBCL) [17]. Yang et al. developed a deep learning system that effectively differentiated metastatic carcinoma from lymphoma in enlarged cervical lymph nodes [18]. Despite these promising advances, the application of radiomics to the specific clinical challenge of distinguishing lymphoma from HNCUP remains largely unexplored.

While Tomita et al. reported preliminary findings on the differentiation of cervical lymphadenopathy using PET/CT features [19], no model has been specifically developed and validated for distinguishing lymphoma from HNCUP in patients with poorly differentiated malignancies. Furthermore, most existing radiomics models rely exclusively on image-derived features, failing to integrate important clinical and metabolic parameters that could synergistically improve diagnostic performance. In particular, quantitative assessment of intratumoral metabolic heterogeneity including the heterogeneity index (HI), which provides unique insights into tumor biology and aggressiveness, remains significantly underutilized in current diagnostic approaches [20]. To the best of our knowledge, no study has investigated the potential of ^18^F-FDG PET/CT radiomics combined with clinical characteristics and metabolic parameters for differentiating lymphoma from HNCUP in patients with poorly differentiated malignancies involving cervical lymph nodes. Therefore, the present study aimed to develop and validate a combined clinical–radiomics nomogram for the accurate differentiation of lymphoma and HNCUP in this diagnostically challenging patient population.

## 2. Materials and Methods

### 2.1. Study Cohort

This retrospective study was conducted in compliance with the Declaration of Helsinki and was approved by the Institutional Review Board of Fudan University Shanghai Cancer Center. The requirement for informed consent was waived owing to the retrospective nature of the study design. To ensure privacy, all images and relevant data were de-identified before analysis and reporting, with no personally identifiable information used. Participants did not receive financial or other material compensation for their involvement.

We initially screened 1242 patients who underwent ^18^F-FDG PET/CT imaging and received a pathological diagnosis of poorly differentiated head and neck cancer between January 2010 and July 2022. Exclusion criteria were as follows. Patients were excluded if they had no pretreatment ^18^F-FDG PET/CT scans. Patients were excluded if PET/CT images were unavailable for reconstruction. This included cases where raw sinogram data could not be re-reconstructed using standardized parameters. Patients were excluded if they had a prior or concurrent diagnosis of a primary malignant tumor. Patients were excluded if their follow-up duration was less than one month. After applying these criteria, 192 patients with clinically and pathologically confirmed as HNCUP or lymphoma were enrolled for further analysis. All variables used in the primary analysis were complete. In the sensitivity analysis converting SUV to SUL, one patient was excluded because body height was unavailable; this patient remained in the primary analysis.

All patients underwent standard diagnostic procedures in accordance with clinical guidelines prior to enrollment. All patients received histopathological confirmation through fine-needle aspiration and/or core needle biopsy. Final diagnoses of lymphoma or HNCUP were established based on comprehensive pathological evaluation including immunohistochemistry. HPV testing via p16 immunohistochemistry and/or HPV in situ hybridization and EBV testing via EBV-encoded RNA in situ hybridization were performed as part of the standard clinical work-up. Patients were classified as HNCUP only if they met three conditions. First, histopathological examination confirmed a malignant neoplasm without features specific to lymphoma. Second, HPV and EBV testing yielded negative or inconclusive results. Third, no primary tumor was identified despite comprehensive clinical and imaging evaluation. Patients classified as lymphoma were diagnosed based on established World Health Organization diagnostic criteria with appropriate immunohistochemical markers.

Clinical characteristics including age, gender, and body mass index (BMI) were collected. Patients were randomly divided into a training group of 134 patients and a test group of 58 patients. This represented a 70% to 30% split. Simple random sampling with a fixed random seed of 42 was used to ensure reproducibility [21]. The randomization was performed using the numpy.random.permutation function in Python (version 3.7; Python Software Foundation, Wilmington, DE, USA; https://www.python.org) with the open-source NumPy library (version 1.21; https://numpy.org).

Follow-up was initiated from the date of pathological diagnosis. The one-month minimum follow-up duration was selected solely as an exclusion criterion to ensure data completeness and diagnostic reliability. It was not used as the basis for final diagnostic confirmation. All included patients had an actual follow-up duration exceeding six months. Diagnostic classifications were established at the time of initial diagnosis. Subsequent follow-up was mainly used to verify clinical outcomes and exclude cases in which a primary tumor might later be identified leading to diagnostic reclassification. For patients with HNCUP, the primary tumor was subsequently identified in some cases during longer follow-up periods. These cases were still classified as HNCUP at the time of initial diagnosis. This time point represents the clinically relevant window for application of the proposed nomogram.

### 2.2. Image Acquisition and Morphological Imaging Assessment

^18^F-FDG was produced in-house using a Siemens Eclipse (RDS111) cyclotron (Siemens Molecular Imaging, Knoxville, TN, USA) coupled with an automated FDG synthesis module NEPTIS (ORA, Neuville, Belgium) at the radiopharmacy of the Department of Nuclear Medicine, Fudan University Shanghai Cancer Center (Shanghai, China). An ^18^F-FDG PET/CT examination was carried out one hour after the intravenous injection of 3.7 MBq/kg (0.1 mCi/kg) ^18^F-FDG in accordance with the patients’ body weight. Images were acquired using two Siemens PET/CT systems. The first system was the Biograph 16 HR (Siemens, Erlangen, Germany). CT acquisition parameters included 120 kV tube voltage, 140 mAs tube current, 5 mm slice thickness and a B31f reconstruction kernel. PET images were reconstructed using the ordered-subset expectation maximization (OSEM) algorithm. Reconstruction settings included 2 iterations, 20 subsets, a Gaussian filter with full width at half maximum of 4 mm, a 168 × 168 matrix and an original voxel size of 4.0 × 4.0 × 2.0 mm^3^. The second system was the Biograph mCT Flow (Siemens, Erlangen, Germany). CT acquisition parameters were identical to those of the Biograph 16 HR system. PET images were also reconstructed using the OSEM algorithm. Reconstruction settings included 2 iterations, 20 subsets, a Gaussian filter with full width at half maximum of 4 mm, a 200 × 200 matrix and an original voxel size of 4.0 × 4.0 × 2.0 mm^3^. Both systems employed continuous bed motion for PET acquisition. Each bed position was scanned for a duration of 2 min. The fused PET/CT images were presented on Syngo workstations (Siemens, Knoxville, Tennessee, USA). Of the 192 enrolled patients, 128 underwent scanning with the Biograph 16 HR system and 64 with the Biograph mCT Flow system. The Biograph 16 HR system included 104 HNCUP cases and 24 lymphoma cases. The Biograph mCT Flow system included 47 HNCUP cases and 17 lymphoma cases.

A nuclear medicine radiologist (with 5–10 years of experience) independently interpreted the PET/CT images without knowledge of the final pathological diagnosis. She documented parameters including morphological features, maximum and mean Standardized Uptake Value (SUV_max_ and SUV_mean_), metabolic tumor volume (MTV), total lesion glycolysis (TLG), and HI. The HI was computed as the ratio of SUV_max_ to SUV_mean_ within the volume of interest (VOI), which reflects the metabolic heterogeneity of the tumor. MTV was delineated using a relative threshold of 41% of the SUV_max_ within the VOI, in accordance with the European Association of Nuclear Medicine guidelines for metabolic volume delineation. TLG was calculated as MTV multiplied by SUV_mean_ within the corresponding thresholded volume. The 41% SUV_max_ threshold was chosen because it provides the best compromise between reproducibility and accuracy for metabolic tumor volume delineation across multiple tumor types.

### 2.3. Image Segmentation

^18^F-FDG PET/CT images in DICOM format were retrieved from the institutional picture archiving and communication system. All segmentations were performed using ITK-SNAP (version 4.0.1; Penn Image Computing and Science Laboratory, University of Pennsylvania, Philadelphia, PA, USA; https://www.itksnap.org) and 3D-Slicer (version 5.2.2; Surgical Planning Laboratory, Brigham and Women’s Hospital, Harvard Medical School, Boston, MA, USA; https://www.slicer.org) by an experienced nuclear medicine physician with more than five years of clinical practice. All segmentations were conducted in a fully manual manner. No semi-automatic thresholding algorithms were used for VOI delineation.

The physician first identified the lesion with the highest SUV_max_ in the head and neck region. The tumor boundary was then manually traced on each axial slice with reference to both PET signal intensity and corresponding CT morphological features [22]. Individual enlarged lesions were delineated separately whenever possible. In cases where adjacent lesions were confluent and could not be clearly separated, the entire involved region was outlined as a single VOI [23].

PET images provide superior delineation of metabolically active tumor regions. Lesion boundaries on CT images may be less distinct in poorly differentiated malignancies. Therefore, volumes of interest initially defined on PET images were transferred to the corresponding co-registered CT images using the software’s built-in rigid registration function. This approach is methodologically sound. PET and CT components of the PET/CT examination are acquired in the same session with the patient in an identical position. The integrated system also applies a standardized automated co-registration algorithm during image reconstruction. The registration accuracy was visually verified by the operating physician for each case to ensure perfect spatial correspondence between the two modalities. A representative example of the segmentation process is provided in Supplementary Figure S1.

### 2.4. Image Standardization and Feature Extraction

CT and PET images inherently differ in spatial resolution and native voxel dimensions. To eliminate this cross-modality variability prior to feature extraction, both CT and PET volumes were resampled to a uniform 1 × 1 × 1 mm^3^ voxel grid using bilinear interpolation [24]. For CT, preprocessing included Hounsfield Unit (HU) normalization and intensity windowing with a window width of 500 and a window level of 50. For PET, all examinations in this retrospective cohort were acquired under our institutional standard clinical protocol, which directly outputs body-weight-normalized SUV (SUV_bw_) as the scanner’s default quantitative metric. SUV_bw_ was calculated as tissue activity concentration divided by injected dose per unit body weight. This fully standardized preprocessing pipeline is designed to maximize the discriminative consistency of all extracted radiomic texture features.

While PERCIST 1.0 guidelines recommend lean-body-mass-normalized SUV (SUL) as the preferred normalization approach to minimize adipose tissue-related quantification bias [25], prospective mandatory SUL calculation at the time of image reconstruction was not feasible for this retrospectively assembled clinical dataset. To rigorously validate the robustness of our SUV_bw_-based workflow, we conducted a dedicated post hoc sensitivity analysis in Section 3.6.

We controlled for inter-scanner quantification variability through a combination of unified acquisition protocols and standardized preprocessing pipelines. All PET/CT scans in this cohort were acquired on two systems from the same vendor, with fully matched acquisition and reconstruction parameters, including identical OSEM algorithm settings, iteration and subset counts, and Gaussian filter full width at half maximum. The preprocessing workflow, which incorporates isotropic voxel resampling, SUV normalization and CT HU intensity standardization, has been previously validated to substantially reduce scanner-driven measurement variability [26].

We deliberately omitted additional ComBat harmonization in this study. ComBat is originally designed to correct for large, heterogeneous batch effects across multi-center datasets with highly divergent imaging protocols [27]. Given our single-vendor setup with only two scanners and fully standardized imaging routines, the residual inter-scanner batch effect after basic preprocessing was expected to be negligible. Applying ComBat on a dataset with only two scanner groups also provides very limited degrees of freedom for stable empirical Bayes estimation, which risks generating unreliable, over-adjusted feature values. This approach aligns with existing radiomics methodology recommendations that standardized imaging protocols paired with basic preprocessing are sufficient for variability correction when inter-scanner differences are minimal [28].

Within the VOI, a total of 3934 quantitative radiomic features were extracted from paired PET and CT images to comprehensively describe the intrinsic phenotypic characteristics of the target lesion. All features were categorized into four standard radiomic families, including first-order statistical features for grayscale intensity distribution, morphological features for tumor geometric properties, texture features derived from four canonical matrix definitions, and filter-based features generated via a series of standard image transforms. The texture feature group was built from the gray-level co-occurrence matrix, gray-level run-length matrix, gray-level size zone matrix and neighboring gray-tone difference matrix. Filtered features were computed across multiple image transforms covering wavelet decomposition, Laplacian of Gaussian, square, logarithmic, and exponential operations to derive multi-resolution representations of the same underlying grayscale, morphological and texture lesion signatures [29].

The full feature-extraction workflow was fully implemented in Python using the open-source PyRadiomics library (version 3.0.1; https://pyradiomics.readthedocs.io, accessed on 23 September 2026). For wavelet decomposition, PyRadiomics adopts the Stationary Wavelet Transform (SWT) via the PyWavelets library (open-source; https://pywavelets.readthedocs.io, accessed on 23 September 2026), with the default Coiflet wavelet of order 1 (coif1). This undecimated, translation-invariant transform preserves all original input spatial dimensions across every decomposed sub-band, without sub-band downsampling. Boundary processing was standardized across all scans: images were pre-padded via numpy.pad() in circular wrap mode to enforce even spatial dimensions, followed by PyWavelets’ native symmetric boundary handling during transform execution. At the default level-1 3D decomposition, this pipeline yields one approximation sub-band and seven independent detail sub-bands.

To systematically validate that our selected wavelet basis delivers robust and generalizable radiomic signatures, we specified a supplementary wavelet sensitivity analysis in the study protocol. In addition to the coif1 wavelet, we included three other wavelet families widely recommended in existing radiomics methodology guidelines. All four wavelet bases were processed under strictly identical conditions, sharing the same SWT decomposition pipeline, preprocessing steps, boundary handling rules, and downstream LASSO feature selection framework.

### 2.5. Radiomic Features Selection and Model Development

To ensure the reproducibility of extracted radiomic features, we performed an intra-observer test–retest reliability analysis. A subset of 30 randomly selected patients underwent independent re-segmentation by the same physician after a minimum washout interval of 4 weeks, blinded to the initial delineation results. For each of the 3934 extracted features, the intra-class correlation coefficient (ICC) was computed using a two-way mixed-effects model for absolute agreement. Features with ICC < 0.75 were considered poorly reproducible and excluded from subsequent analysis.

Feature selection was performed next to identify robust and non-redundant features. All features were standardized using z-score normalization with a mean of zero and a standard deviation of one before feature selection. This ensured equal contribution of features with different measurement scales. The z-score normalization parameters (mean and standard deviation) were calculated exclusively on the training cohort and subsequently applied to the test cohort to avoid information leakage. The extracted features were divided into two single-modality feature sets and one combined multimodality feature set. The single-modality sets included a PET feature set and a CT feature set. The combined set included all PET and CT features.

The least absolute shrinkage and selection operator (LASSO) method with ten-fold cross-validation was utilized for feature selection. The optimal lambda value was determined by the minimum binomial deviance criterion during cross-validation. A lambda value of 0.032 was used for the combined PET/CT feature set. Features with non-zero coefficients at the optimal lambda value were retained for subsequent model development.

We did not apply a prior Spearman correlation-based filtering step before LASSO analysis. This decision was based on two key methodological considerations. First, LASSO employs L1 regularization. This regularization inherently addresses feature redundancy by selecting representative features from highly correlated groups while shrinking coefficients of other features to zero. This property has been extensively validated in the radiomics literature. The landmark study by Aerts et al. [30] applied LASSO directly to 440 radiomic features without prior correlation filtering. Parmar et al. [31] similarly applied LASSO directly to high-dimensional feature sets and found it among the most stable and effective feature selection approaches. Both studies achieved robust feature selection without the two-step correlation filtering and LASSO approach. Second, prior correlation filtering may be counterproductive and risks discarding informative features. Weakly correlated features that would be retained by a lenient correlation threshold may still carry complementary predictive value that LASSO can exploit. A strict threshold may erroneously eliminate such features. In the context of our high feature-to-sample ratio of 3934 features to 192 samples, aggressive pre-filtering risks exacerbating information loss by prematurely discarding features that could contribute to model performance.

Following feature selection, the retained radiomic features were fed into eight representative machine learning classifiers for comparative performance evaluation: logistic regression (LR), k-nearest neighbors, support vector machine, multi-layer perceptron, light gradient boosting machine, random forest, extreme gradient boosting and extra trees. All models underwent hyperparameter optimization via grid search with 10-fold cross-validation. The predefined criteria for selecting the final optimal classifier were set as follows: high interpretability, robust performance under small-sample constraints, native calibrated probability output for nomogram construction, and consistent performance across different data splits. The radiomics score (Rad_score) for each patient was defined a priori as the linear combination of selected features weighted by their corresponding LASSO-derived coefficients.

This study presented an inherent class imbalance, with a lymphoma-to-HNCUP case ratio of approximately 1:3.7 (21% lymphoma cases). Instead of adopting conventional imbalance correction strategies including the Synthetic Minority Over-Sampling Technique (SMOTE), oversampling, or class weighting, we addressed the class imbalance issue by integrating clinical variables into radiomics models. This strategy was adopted based on recent methodological evidence that conventional imbalance correction approaches may compromise the calibration performance of clinical prediction models. Previous studies have systematically verified the adverse effects of traditional correction methods. Specifically, van den Goorbergh et al. [32] reported that resampling methods cause strong miscalibration while failing to improve discrimination of patient outcome status. Carriero et al. [33] validated this conclusion across multiple machine learning algorithms. Integration of clinical variables resolved sensitivity limitations without additional data manipulation. Ligero et al. [34] further indicated that resampling strategies tend to undermine radiomics model performance. Given that our nomogram was developed for individualized risk probability estimation, retaining the original cohort event rate is essential to guarantee reliable and well-calibrated predictive results.

### 2.6. Development of the Clinical–Radiomics Signature and Nomogram

Clinical characteristics including age, gender and BMI and ^18^F-FDG PET/CT parameters including SUV_max_, SUV_mean_, MTV, TLG and HI were compared between HNCUP and lymphoma groups in both the training and test cohorts. An HI cutoff value of 2.37 was determined using Youden’s index based on the receiver operating characteristic (ROC) curve derived from the training cohort. HI was the only continuous variable for which a binary cutoff was established. This decision was based on three key considerations. First, HI is a dimensionless ratio reflecting metabolic heterogeneity that is more amenable to binary stratification than absolute metabolic measures. Second, it demonstrated the strongest discriminative ability among all continuous PET/CT parameters in univariate analysis. Third, converting HI to a binary variable simplified the clinical model and nomogram, facilitating point-of-care clinical application.

The selected independent predictive variables were used to construct a clinical model using LR. Backward elimination based on the Akaike information criterion retained only gender and HI in the final multivariate clinical model. MTV and TLG were not retained simultaneously. SUV_max_ and SUV_mean_ were also not retained simultaneously. No significant multicollinearity was present in the final model, as no collinear variable pairs were retained together [35,36]. The clinical–radiomics model was constructed by combining the selected clinical variables with the ^18^F-FDG PET/CT radiomics signature. The nomogram was developed based on the predicted probabilities from the combined clinical–radiomics LR model.

The nomogram score calculation follows a transparent step-by-step procedure. First, the clinical signature is computed as one divided by one plus the exponential of the negative sum of the intercept, the product of the gender coefficient and gender value, and the product of the HI coefficient and binary HI value. Gender is coded as one for female and zero for male. Binary HI is coded as one if HI exceeds 2.37 and zero otherwise. Second, the radiomics signature is computed as one divided by one plus the exponential of the negative radiomics score. The radiomics score represents the linear combination of selected radiomic features weighted by their respective LASSO coefficients. Third, both the clinical signature and radiomics signature range from zero to one and map linearly to their respective axes with scores ranging from zero to 100 points. The total points are calculated as the sum of the two axis scores. This total score is then projected onto the risk axis to obtain the individualized probability of lymphoma. A worked example illustrating this calculation process is provided in Section 3.3.

### 2.7. Nomogram Evaluation

The diagnostic performance of the radiomics, clinical, and clinical–radiomics nomogram was evaluated using AUCs with a 95% confidence interval (95% CI). Accuracy, sensitivity, specificity, positive predictive value (PPV), negative predictive value (NPV), and F1 score were evaluated in both the training and test sets. Calibration curves were generated, and the Hosmer–Lemeshow (HL) test, and calibration slope and intercept (where a slope of 1 and intercept of 0 indicate perfect calibration). Decision curve analysis (DCA) was performed to estimate the clinical utility of the clinical–radiomics nomogram.

### 2.8. Statistical Analysis

Clinical data and PET/CT parameters were compared between HNCUP and lymphoma. Continuous variables were compared using the Mann–Whitney U test, and categorical variables were compared using the chi-square test (Fisher’s exact test was applied where expected cell counts were <5). Multivariate logistic regression analysis was conducted to identify the independent predictive parameters. The statistical significance between the AUCs of three models was tested using DeLong’s method. The HL test was conducted to evaluate the goodness of fit of the model. All statistical tests were two-sided, and a *p*-value less than 0.05 was considered significant. Statistical analyses were performed using IBM SPSS Statisticsb (version 26.0; IBM Corp., Armonk, NY, USA), R software (version 4.3.0; R Foundation for Statistical Computing, Vienna, Austria), and Python (version 3.7; Python Software Foundation, Wilmington, DE, USA).

## 3. Results

### 3.1. Participant Characteristics

The patient inclusion and exclusion workflow is summarized in Figure 1. Baseline clinical characteristics of the enrolled cohort are detailed in Table 1. A total of 192 consecutive patients were ultimately included in the present study, which was randomly split into a training cohort of 134 patients and an independent test cohort of 58 patients. The median age was 59 years (range: 22–85 years) in the training cohort and 60 years (range: 26–78 years) in the test cohort. The overall cohort comprised 151 (78.6%) patients with HNCUP and 41 (21.3%) patients with lymphoma, yielding a class imbalance ratio of approximately 3.7:1. This distribution closely reflects the real-world clinical prevalence of the two conditions in routine head and neck oncological practice. Subgroup analysis by sex showed that 17.9% (27/151) of HNCUP patients and 53.7% (22/41) of lymphoma patients were female. No statistically significant inter-cohort differences were identified in any baseline demographic or clinical variable between the training and test groups.

Lymphoma subtype distribution was analyzed in the 41 confirmed lymphoma cases. DLBCL was the most common subtype with 31 cases. Hodgkin lymphoma accounted for four cases. Follicular lymphoma accounted for two cases. T-cell lymphoma accounted for one case. The remaining three cases were classified as other rare subtypes. Gender, SUV_max_, SUV_mean_, TLG and HI showed statistically significant differences between HNCUP and lymphoma groups in both the training and test cohorts. All *p* values were less than 0.05. Multivariate logistic regression analysis identified two independent predictors for distinguishing between the two pathological types. Female gender was associated with an odds ratio (OR) of 8.793 (95% CI: 2.801–27.607). The *p* value was less than 0.001. An HI value greater than 2.37 was associated with an OR of 5.762 (95% CI: 1.422–23.348). The *p* value was 0.014. Detailed results of the multivariate analysis are presented in Table 2.

Notably, the 95% CIs for these independent predictors (e.g., Gender OR 95% CI: 2.801–27.607; HI OR 95% CI: 1.422–23.348) reflect the limited statistical power arising from the small lymphoma sample size (*n* = 41), and the point estimates of these ORs should be interpreted with appropriate caution. The direction of these associations is consistent with established epidemiological and biological evidence. However, the magnitude of these effects should be interpreted with caution. Further validation in larger independent cohorts is required to confirm these findings.

### 3.2. Results of the Selected Radiomics Signatures

Three distinct radiomic signatures were generated after LASSO feature selection. The PET-only signature contained 12 radiomic features, the CT-only signature contained 9 radiomic features, and the combined PET/CT signature consisted of 21 features derived from both modalities.

The selected features across all three signatures spanned morphological descriptors, first-order statistical metrics, and second-order textural features, which were extracted from original images, wavelet-transformed images and other filter-derived transformed images. The full inventory of selected features and their corresponding LASSO regression coefficients for the two single-modality signatures are reported in Supplementary Table S1. The feature composition, individual LASSO coefficient values and relative weight distribution of the 21-feature combined PET/CT signature are visualized in Figure 2.

### 3.3. Performance of LR-Based Radiomics Models

Across the complete panel of eight evaluated machine learning models, LR yielded the minimal train–test performance gap and the most consistent discriminative outputs across all nested ten-fold cross-validation splits, fully satisfying all four predefined classifier selection criteria. Full performance metrics for all eight competing models are documented in Supplementary Tables S2–S4. LR was therefore designated as the optimal final classifier.

The discriminative performance of these three LR-based radiomics models in both the training and independent internal test cohorts is comprehensively detailed in Table 3. For the standalone CT signature, the LR model achieved an AUC of 0.791 (95% CI: 0.699–0.883) in the training cohort and 0.817 (95% CI: 0.703–0.932) in the test cohort. The standalone PET signature yielded a training cohort AUC of 0.828 (95% CI: 0.738–0.918) and a test cohort AUC of 0.692 (95% CI: 0.521–0.863). The combined PET/CT radiomics signature delivered the highest performance, with a training cohort AUC of 0.916 (95% CI: 0.855–0.976) and a test cohort AUC of 0.812 (95% CI: 0.682–0.942). Detailed ROC curves for all three LR models in both cohorts are presented in Figure 3.

Notably, in the PET/CT radiomics LR model, sensitivity decreased substantially from 0.857 in the training cohort to 0.538 in the test cohort, while specificity remained high at 0.911. This pattern indicates that the model maintained excellent ability to correctly identify HNCUP cases but showed reduced ability to detect lymphoma cases in the independent test cohort. Several factors may contribute to this observed sensitivity drop. Class imbalance with only 21 percent lymphoma cases may have biased the model toward the majority class. The heterogeneous nature of lymphoma subtypes in our cohort may also have affected model generalizability. The relatively small number of lymphoma cases in the test cohort further limited statistical power for sensitivity assessment. This observation underscores the importance of combining radiomics features with clinical variables to achieve more robust and balanced diagnostic performance.

### 3.4. Performance of Clinical–Radiomics Nomogram

The diagnostic performance of the three constructed models, namely the clinical nomogram, radiomics nomogram and integrated clinical–radiomics nomogram, is summarized in Table 4 and visualized in Figure 4. In the training cohort, the clinical–radiomics nomogram yielded the highest discriminative ability with an AUC of 0.924 (95% CI: 0.868–0.980), followed by the standalone PET/CT radiomics nomogram (AUC = 0.916, 95% CI: 0.855–0.976). The clinical nomogram exhibited the lowest discriminative performance (AUC = 0.723, 95% CI: 0.624–0.823). The clinical–radiomics nomogram achieved well-balanced sensitivity (0.821) and specificity (0.896), alongside the highest overall accuracy of 0.881 across all three evaluated models. In the test cohort, the clinical–radiomics nomogram maintained robust generalization performance with an AUC of 0.912 (95% CI: 0.837–0.987). Notably, its high sensitivity of 0.923 effectively compensated for the sensitivity decline observed in the standalone radiomics model. These consistent results confirm that integrating curated clinical variables with radiomic features substantially improves the generalization robustness and predictive balance of the final diagnostic model.

Notably, the clinical nomogram delivered a higher AUC in the test cohort than in the training cohort, an apparently counterintuitive phenomenon that can be explained by three key factors. First, the clinical model incorporates only two predictors, gender and HI, leading to extremely low model complexity and a negligible risk of overfitting. Second, the HI indicator presented more significant intergroup differences in the test cohort. In the test cohort, the median HI was 2.7 for lymphoma cases and 2.2 for HNCUP cases (difference = 0.5, *p* = 0.006), while the training cohort showed a smaller median HI difference (2.6 vs. 2.3, difference = 0.3, *p* = 0.001). As an independent predictive factor with an OR of 5.762 for HI > 2.37, the enhanced intergroup separation of HI primarily contributed to the elevated test set AUC of the clinical model. Third, the relatively small size of the test cohort inherently introduced sampling uncertainty into AUC estimation, as reflected by the wide 95% CI of 0.799–1.000. This improvement in performance should therefore be interpreted with appropriate caution. Despite the elevated test-set AUC, the clinical model only achieved a positive predictive value of 0.733, which remained substantially lower than that of the clinical–radiomics nomogram, further verifying the superior comprehensive performance of the combined model.

A representative clinical case is presented to illustrate the individualized scoring workflow and practical clinical application of the proposed nomogram (Figure 5). For a female patient with an HI value of 2.5 (binary HI variable = 1, as defined by the optimal cutoff of 2.37) and a radiomics score of 0.8, the point contribution of each predictor was first obtained by mapping the variable value to the corresponding point scale on the nomogram. The continuous radiomics score was converted to a predictive probability via standard logistic transformation, yielding a value of approximately 0.690, which corresponded to 69 points on the radiomics signature axis. Points from all clinical variables and the radiomics signature were summed to generate the total nomogram score. Finally, the individualized lymphoma risk probability was derived by projecting this total score onto the final risk probability axis of the nomogram. A web-based interactive calculator is currently under development to support convenient point-of-care deployment of this nomogram in routine oncological practice.

### 3.5. Results of Statistical Analysis, Calibration, and Discrimination

Statistical comparisons of model discrimination were conducted using DeLong’s test. In the training cohort, the nomogram demonstrated a significantly higher AUC compared to the clinical signature (*p* = 3.755 × 10^−4^). However, no significant difference in AUC was observed between the nomogram and the radiomics signature (*p* = 0.134). In the test cohort, the discriminatory performance of the nomogram was statistically superior to that of the radiomics signature (*p* = 0.026), while no significant difference was found with the clinical signature (*p* = 0.911).

Calibration of the predictive models was quantitatively evaluated using calibration curves, HL test, calibration slope, and intercept (Figure 6). The nomogram achieved satisfactory calibration in the training cohort, which closely matched the ideal 45-degree reference line, with a calibration slope of 1.197, an intercept of 0.239, and a non-significant HL test result (*p* = 0.261). In the test cohort, however, mild-to-moderate miscalibration was observed, predominantly for high-risk predictions. The test-set calibration slope was 2.364 (intercept = 1.875), pointing to model overconfidence where predicted probabilities are compressed across the low-to-mid range and rise sharply for high-risk patients; the corresponding HL test yielded *p* = 0.078, indicating marginal deviation from perfect calibration. Comparative analysis showed that the radiomics signature maintained stable cross-cohort calibration (test set *p* = 0.219), whereas the standalone clinical signature exhibited poor calibration in the test set (*p* = 0.005). This test-set miscalibration was primarily attributed to the small test cohort with limited lymphoma cases, cohort-specific subtype differences, and moderate class imbalance, which rendered high-risk probability estimation unstable. To rectify the miscalibration bias, we conducted exploratory post hoc recalibration using Platt scaling (logistic calibration) and isotonic regression. Platt scaling improved the calibration slope from 2.364 to 2.222 (intercept = 1.685), while isotonic regression improved the slope to 1.506 (intercept = 0.882); they did not alter the primary model conclusions. Notably, these recalibration analyses are exploratory only, and recalibrated probabilities were not adopted as the primary model outputs. Given the limited test cohort size, these corrective results should be interpreted with caution due to potential optimistic bias.

To evaluate the clinical net benefit of each model, DCA was performed (Figure 7). In the training cohort, the net benefit curves of the nomogram and the radiomics-only model were nearly indistinguishable across most threshold probabilities, indicating that adding clinical parameters provides limited incremental benefit within the training data, consistent with the DeLong’s test results (*p* = 0.134). In the test cohort, the clinical–radiomics nomogram demonstrated optimal net benefit within the 5–60% threshold range, outperforming both the radiomics-only model and the treat all/treat none reference strategies. The clinical model showed higher net benefit at thresholds >60%; however, this high-threshold range corresponds to a clinical scenario where clinicians would only initiate lymphoma chemotherapy when they are very certain of the diagnosis, which has limited practical value in the diagnostic gray zone where the nomogram is intended to be applied. Furthermore, the clinical model’s poor calibration (HL test *p* = 0.005) undermines the reliability of its apparent net benefit advantage at high thresholds. The erratic behavior of the radiomics model curve in the 0.4–0.8 threshold range, including a drop below zero, likely reflects statistical instability driven by the small test sample size (*n* = 58) rather than a genuine clinical characteristic. These DCA results should be interpreted with caution, acknowledging the inherent instability of net benefit estimates in small samples, and future large-sample multi-center studies are needed to confirm these findings.

### 3.6. Sensitivity and Subgroup Analyses for Robustness Evaluation

To assess whether model discrimination is affected by the choice of SUV normalization, we performed a dedicated sensitivity analysis. Volumes from 191 of 192 patients were converted from SUV_bw_ to SUL, and all radiomic features were re-extracted with identical PyRadiomics settings. Per-feature Spearman correlation between the two normalizations was high for most features (median r = 0.97; 85% of features r > 0.80), and the score was strongly concordant (r = 0.86 in the training and r = 0.92 in the test). In a paired re-analysis on the identical patients using the LASSO-selected features, the nomogram test AUC was 0.912 under SUV_bw_ and 0.877 under SUL, with both models retaining strong discrimination (Supplementary Table S5).

To verify model performance stability considering the enrolled population’s wide BMI range, we performed a post hoc BMI subgroup analysis. Patients were stratified into underweight/normal, overweight, and obese, and model discriminative performance across subgroups was compared (Supplementary Table S6). In the training cohort, the model had stable discriminative ability across all BMI strata (AUCs ≥ 918). Test cohort subgroup analysis was attempted, but each BMI stratum had extremely limited lymphoma-positive cases, so results require cautious interpretation due to small-sample. These results further confirmed stable model discrimination across all BMI tertiles, along with a negligible Spearman correlation between BMI and the final radiomics score, demonstrating that SUV_bw_ normalization did not introduce systematic quantification bias in the present study cohort.

To evaluate potential scanner-related systematic bias, patients were stratified by PET/CT scanner type. No significant inter-scanner differences in Rad_score (*p* = 0.100) or predicted probability (*p* = 0.849) were detected in the training cohort. Significant differences were observed in the test cohort (*p* = 0.032 and *p* = 0.044), but the Biograph mCT Flow subgroup contained only nine patients, so these *p*-values most likely reflect random sampling fluctuation rather than true scanner bias. Baseline clinical and PET parameters were balanced between scanner groups (all *p* > 0.05, Supplementary Table S7). Collectively, the standardized preprocessing pipeline largely mitigated scanner-related systematic bias, although test-set subgroup comparisons require cautious interpretation.

As an exploratory sensitivity analysis, we evaluated whether findings depend on the wavelet basis. Wavelet sub-band features were re-extracted using db2, sym3, and bior2.2 at the same decomposition level, holding all non-wavelet features and the LASSO-selected coefficient set constant. The resulting models yielded near-identical performance across all four bases (test AUC differences within 0.02; Supplementary Table S8), indicating that the discriminative wavelet features are not specific to coif1. We retained coif1 given its near-symmetry and common use in PET radiomics. Among the 21 features retained in the final signature, 13 (61.9%) were wavelet-derived, underscoring their contribution to the model.

## 4. Discussion

To the best of our knowledge, this is the first study to develop and validate a clinical–radiomics nomogram based on ^18^F-FDG-PET/CT for differentiating lymphoma from HNCUP in patients with poorly differentiated malignancies involving cervical lymph nodes. Our results suggest that the integration of radiomics signatures with key clinical parameters may improve diagnostic accuracy compared to models based solely on radiomics features or clinical variables alone. The clinical–radiomics nomogram achieved excellent discrimination performance with AUCs of 0.924 and 0.912 in the training and test cohorts, respectively. However, as a single-center retrospective study with a relatively small sample size, these findings should be considered preliminary and require external validation in larger, multi-center cohorts.

The superior performance of our combined model highlights the complementary value of radiomics and clinical information. Radiomic features extracted from PET/CT images capture subtle tumor characteristics beyond conventional quantitative parameters. Clinical variables provide essential contextual information about individual patients. In our study, gender emerged as a strong independent predictor. Female patients were significantly more likely to have lymphoma (OR = 8.793, 95% CI: 2.801–27.607). This finding is supported by epidemiological data indicating a higher incidence of specific lymphoma subtypes in females [37]. The HI, a quantitative measure of intratumoral metabolic variation, also proved valuable as an independent predictor (OR = 5.762 for HI > 2.37, 95% CI: 1.422–23.348). This underscores the importance of tumor heterogeneity in distinguishing between these pathologically distinct entities, as lymphomas often exhibit different metabolic patterns compared to epithelial carcinomas [38]. Importantly, the wide 95% confidence intervals for these ORs reflect the limited statistical power arising from the small lymphoma sample size. These wide intervals indicate substantial uncertainty in the point estimates, and the effect sizes should be interpreted with appropriate caution. This observation also reflects, in part, the biological heterogeneity within the lymphoma group, which comprised multiple histological subtypes (DLBCL, Hodgkin lymphoma, follicular lymphoma, and T-cell lymphoma). Future studies with larger, subtype-stratified cohorts are needed to obtain more precise estimates of the predictive contributions of these clinical variables.

A notable finding in our study is the decline in sensitivity of the radiomics-only model in the test cohort. Sensitivity decreased from 0.857 in the training cohort to 0.538 in the test cohort. In contrast, the clinical–radiomics nomogram sustained a high sensitivity of 0.923. Several factors may contribute to this observed sensitivity drop. Class imbalance with only 21 percent lymphoma cases may have biased the model toward the majority class. The heterogeneous nature of lymphoma subtypes in our cohort may also have affected model generalizability. The relatively small number of lymphoma cases in the test cohort further limited statistical power for sensitivity assessment.

Regarding the calibration performance, the test set calibration slope of 2.364 warrants careful interpretation. This over-confidence pattern in the high-probability range is likely driven by the small number of lymphoma cases in the test set, which results in sparse data points in the upper portion of the calibration curve, making it highly sensitive to individual predictions. The class imbalance (approximately 21% lymphoma) further amplifies this effect. Post hoc calibration using Platt scaling and isotonic regression improved the calibration slope to 2.222 and 1.506, respectively, without affecting discrimination. However, calibration correction on the test set itself introduces optimistic bias, and proper calibration would require a separate calibration dataset in future prospective studies.

We chose not to apply class imbalance correction methods such as SMOTE. This decision was based on evidence that such corrections impair calibration in clinical prediction models. Resampling methods also often degrade radiomics model performance. Instead, integration of clinical variables including gender and heterogeneity index effectively addressed the sensitivity concern. A test set sensitivity of 0.923 was achieved without distorting the real-world prevalence distribution. This approach aligns with recommendations for clinical prediction models to preserve true event rates for well-calibrated risk estimates.

Radiomics has demonstrated utility for differential diagnosis across multiple clinical scenarios. Previous studies have applied radiomics to differentiate renal cell carcinoma from renal lymphoma [39], DLBCL from invasive ductal carcinoma [40], recurrence from inflammation in nasopharyngeal carcinoma [41], autoimmune pancreatitis from pancreatic ductal adenocarcinoma [42,43], and to characterize solitary pulmonary nodules [44,45]. These studies collectively support the broader validity of the radiomics approach for resolving challenging differential diagnoses. They also highlight the importance of developing disease-specific models tailored to individual clinical questions.

The radiomic features selected in this study may reveal intrinsic biological differences between lymphoma and HNCUP. Features incorporated into the final model include textural features derived from wavelet transforms. In our analysis, these wavelet transforms employed the coif1 with symmetric boundary padding and single-level decomposition, generating eight sub-bands. These sub-bands capture heterogeneity patterns at different spatial frequencies and orientations within the tumor. This likely reflects fundamental distinctions in cellular arrangement, microenvironment architecture, or the distribution of metabolic activity between the two entities. Morphological features may be associated with tumor growth patterns. First-order statistical features may reflect the uniformity of metabolic intensity within the lesion. As detailed in Section 3.6, we re-extracted wavelet sub-band features using db2, sym3, and bior2.2, holding all non-wavelet features constant. The resulting models yielded near-identical performance across all four bases, indicating that discriminative wavelet features are not specific to coif1. Future large-scale studies should systematically benchmark additional wavelet families and decomposition levels to determine the optimal basis.

Our lymphoma cohort encompassed multiple histological subtypes. These include DLBCL, Hodgkin lymphoma, follicular lymphoma and T-cell lymphoma. Different lymphoma subtypes are known to exhibit distinct metabolic profiles. DLBCL typically demonstrates higher FDG avidity than indolent subtypes such as follicular lymphoma. However, the clinical objective of our model is binary classification of lymphoma versus HNCUP rather than subtype differentiation. From a clinical perspective, the critical decision point is whether the patient has lymphoma or HNCUP. Lymphoma warrants lymphoma-specific chemotherapy. HNCUP requires primary tumor investigation and site-specific treatment. This distinction applies regardless of the specific lymphoma subtype. In this context, inclusion of multiple subtypes may actually enhance the generalizability of the model for this binary task. The model learns discriminative features that distinguish lymphoma as a broader category from HNCUP rather than features specific to a single subtype. Nevertheless, we acknowledge that heterogeneous metabolic profiles across subtypes may introduce additional variability in radiomic features. Future studies with larger samples should consider subtype-specific analyses.

The clinical–radiomics nomogram offers several practical advantages for managing patients with poorly differentiated malignancies involving cervical lymph nodes. First, it provides a non-invasive tool to assist in differential diagnosis when pathological samples are limited or inconclusive, potentially reducing the need for repeated biopsies. Second, earlier and more accurate differentiation between lymphoma and HNCUP helps guide appropriate treatment strategies. Chemotherapy is the primary treatment for lymphoma. A comprehensive search for the primary lesion and subsequent tailored therapy is recommended for HNCUP. Third, the nomogram’s user-friendly interface allows clinicians to estimate individual patient probabilities quickly at the point of care. It is important to emphasize that the proposed nomogram is intended as a preliminary, exploratory adjunctive tool. It is designed for the specific clinical scenario where conventional diagnostic work-up has yielded inconclusive results. The nomogram is not designed to replace standard diagnostic procedures. It also does not serve as a primary tumor detection or staging tool.

Our findings should be interpreted in light of several limitations. First, this study was a single-institution retrospective investigation with a relatively limited sample size, particularly for lymphoma cases, which may compromise the stability of feature selection and model estimation, as reflected in the wide CIs of independent predictors. The inherent class imbalance of the cohort negatively affected the sensitivity of the radiomics-only model, while the small test-set lymphoma population contributed to unsatisfactory calibration and unstable DCA net benefit estimates. Although post hoc Platt scaling and isotonic regression improved calibration performance, these test-set adjustments may introduce optimistic bias. We mitigated these biases via rigorous cross-validation, but external validation in large, multicenter prospective cohorts with dedicated calibration datasets is still required to confirm model generalizability and robustness. Second, image segmentation and feature selection workflows have room for further optimization. All tumor delineations were initially performed by a single physician, precluding comprehensive evaluation of inter-observer variability. Future studies should incorporate multi-reader inter-observer ICC validation to further strengthen generalizability. In addition, our feature selection pipeline relied on LASSO regularization without prior correlation-based redundancy filtering. Although L1 regularization inherently handles feature collinearity and this strategy is well-supported by the existing radiomics literature, more multi-step feature screening frameworks may further improve the independence and stability of selected features. Future studies should adopt multi-reader segmentation protocols and automated segmentation algorithms to enhance overall reproducibility. Third, the current model has constrained applicability and incomplete variable inclusion. The nomogram was exclusively developed for patients with cervical nodal poorly differentiated malignancies, and its performance for other anatomical sites or lymphoma subtypes remains unclear. Furthermore, only basic clinical variables were incorporated, while retrospective data unavailability precluded the inclusion of auxiliary biomarkers (e.g., LDH, sIL-2R, and leukocyte counts). In addition, PET quantification adopted SUV_bw_ rather than the PERCIST 1.0-recommended SUL. In a post hoc sensitivity analysis, we converted SUV_bw_ to SUL using the Janmahasatian formula and re-extracted all radiomic features. The nomogram test AUC remained high under both normalizations (approximately 0.90), with a modest decrease of ≈0.03 under SUL. Nonetheless, SUV_bw_ may introduce subtle bias across wide BMI ranges, and future prospective studies should implement standardized SUL normalization per PERCIST 1.0 guidelines. Fourth, residual inter-scanner variability cannot be completely excluded despite unified imaging protocols and standardized preprocessing. All images were acquired on two same-vendor scanners with matched acquisition and reconstruction parameters, and our preprocessing workflow substantially reduced scanner-driven heterogeneity. Subgroup analyses showed no inter-scanner discrepancies in the training set, while significant test-set differences were most likely caused by the limited sample size of the Biograph mCT Flow subgroup. We did not apply ComBat harmonization owing to insufficient batches for stable empirical Bayes estimation; future multicenter studies with richer scanner batches are warranted for more rigorous batch effect correction.

Despite these limitations, our study makes several important contributions to the field. We have demonstrated that integrating quantitative radiomic features with key clinical parameters may improve differentiation of lymphoma from HNCUP in diagnostically challenging cases. The developed nomogram provides a practical tool that could support diagnostic decision-making pending external validation. Future research should focus on three key areas. First, prospective multi-center validation of the nomogram in larger patient cohorts. Second, exploration of the biological underpinnings of the identified radiomic features. Third, evaluation of the incremental value of incorporating serum biomarkers and molecular profiling data into the model.

## 5. Conclusions

The clinical–radiomics nomogram based on ^18^F-FDG-PET/CT represents a promising but preliminary non-invasive approach for distinguishing lymphoma from HNCUP in patients with poorly differentiated malignancies involving cervical lymph nodes. By combining the strengths of advanced imaging analysis with clinical insights, this model shows potential to aid diagnostic decision-making in this clinically challenging scenario. However, this is a single-center retrospective study. These findings should be considered exploratory given the known limitations including class imbalance, limited sample size (particularly for lymphoma), wide 95% CIs for independent predictors that indicate substantial uncertainty in effect-size estimates, and lack of external validation. Prospective, multi-center validation with larger cohorts is essential before any clinical implementation.

## Figures and Tables

**Figure 1:**
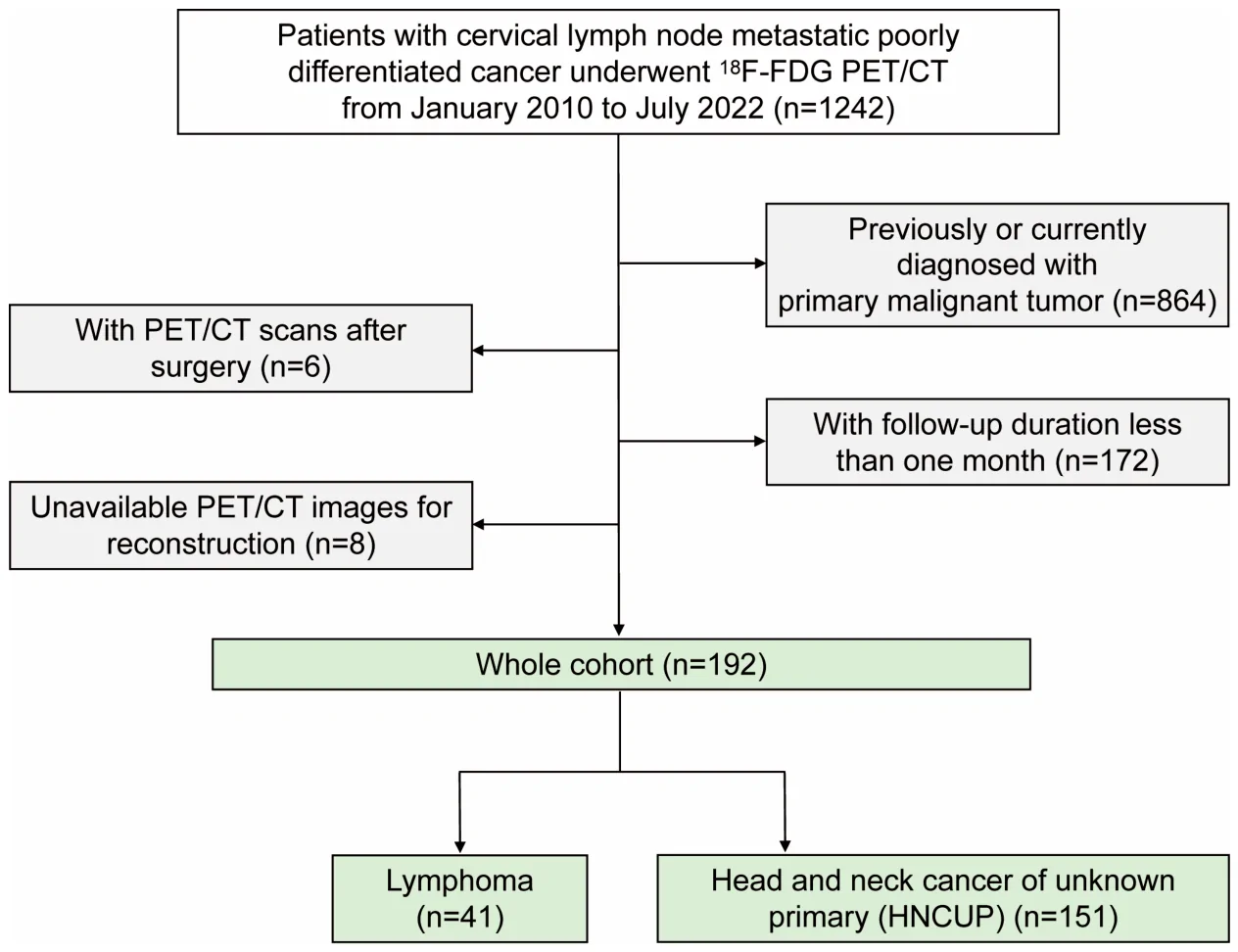
Flowchart of study patient selection.

**Figure 2:**
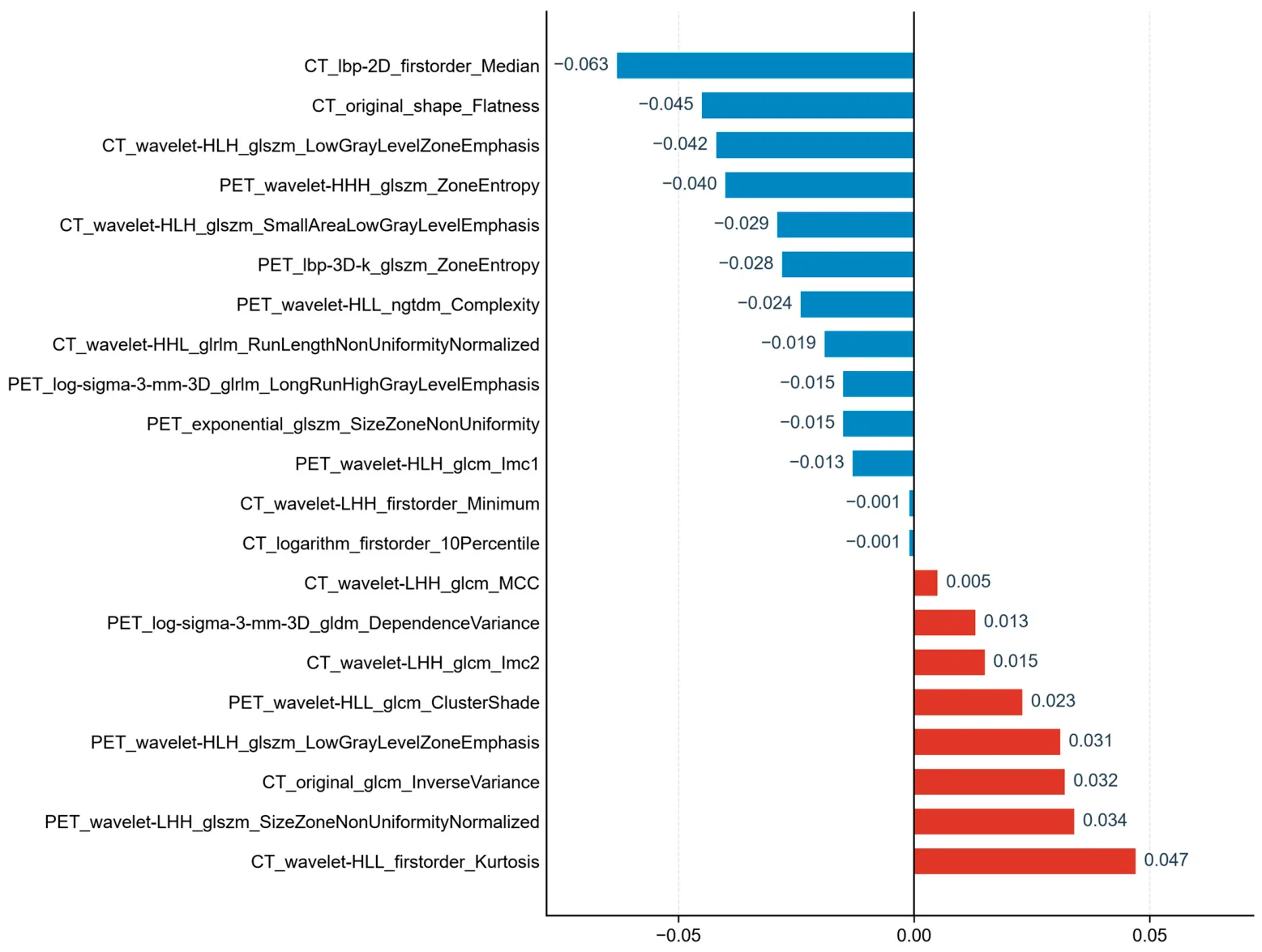
Relative LASSO coefficient weights of the 21 radiomic features in the final combined PET/CT radiomics signature. Red bars represent positive LASSO coefficients, and blue bars represent negative coefficients.

**Figure 3:**
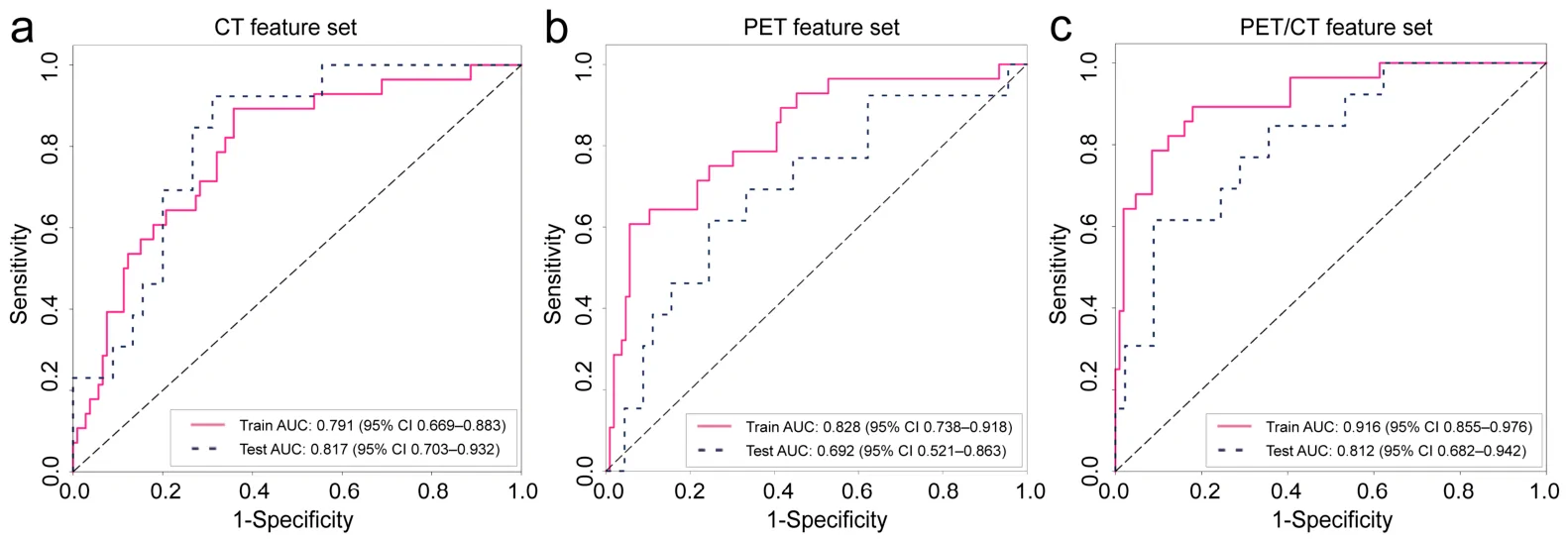
ROCs of the LR-based radiomics models in training and test cohorts: (**a**) represents the CT radiomics model, (**b**) represents the PET radiomics model, (**c**) represents the PET/CT radiomic model. The diagonal dashed line indicates the reference line for random classification (AUC = 0.5).

**Figure 4:**
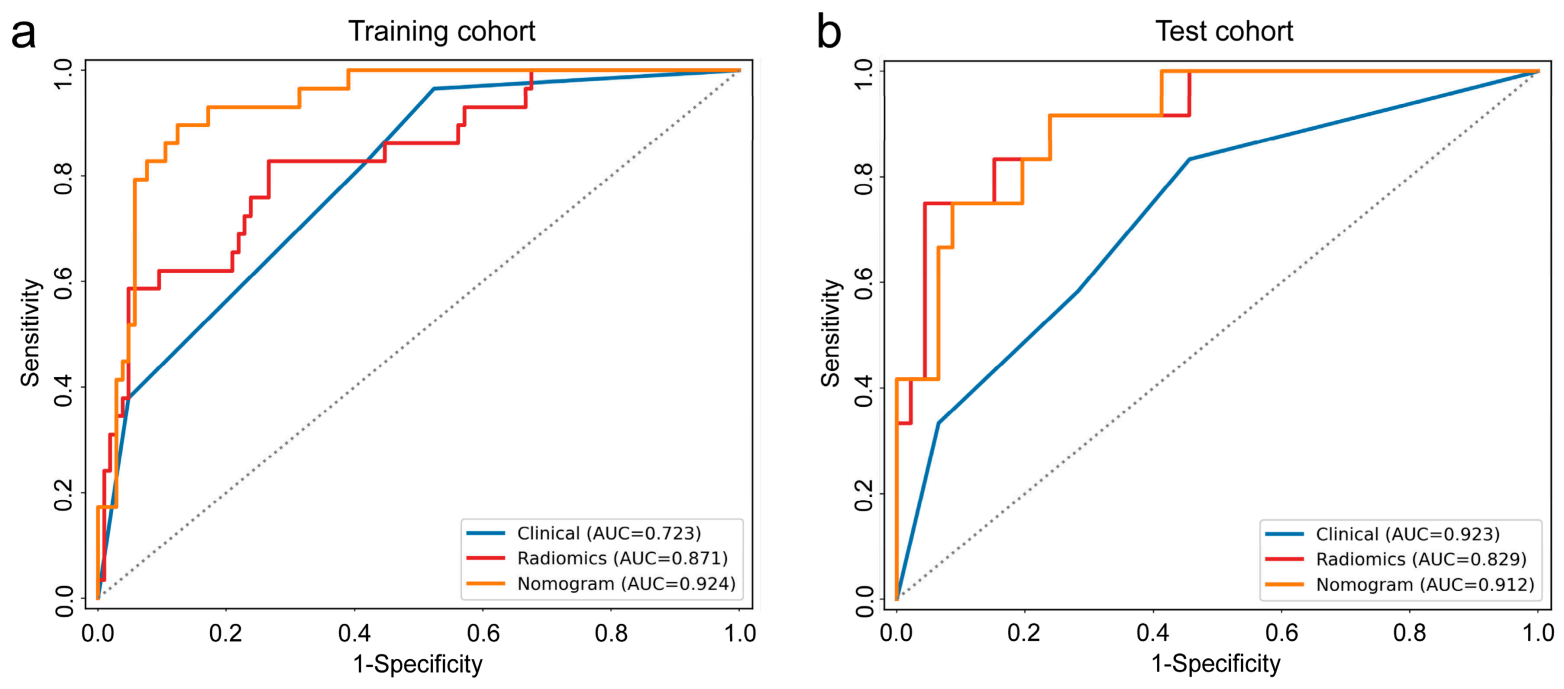
ROC curves of the clinical nomogram, radiomics nomogram, and clinical–radiomics nomogram in training and test cohorts: (**a**) represents the training cohort, (**b**) represents the test cohort. The diagonal dashed line indicates the reference line for random classification (AUC = 0.5).

**Figure 5:**
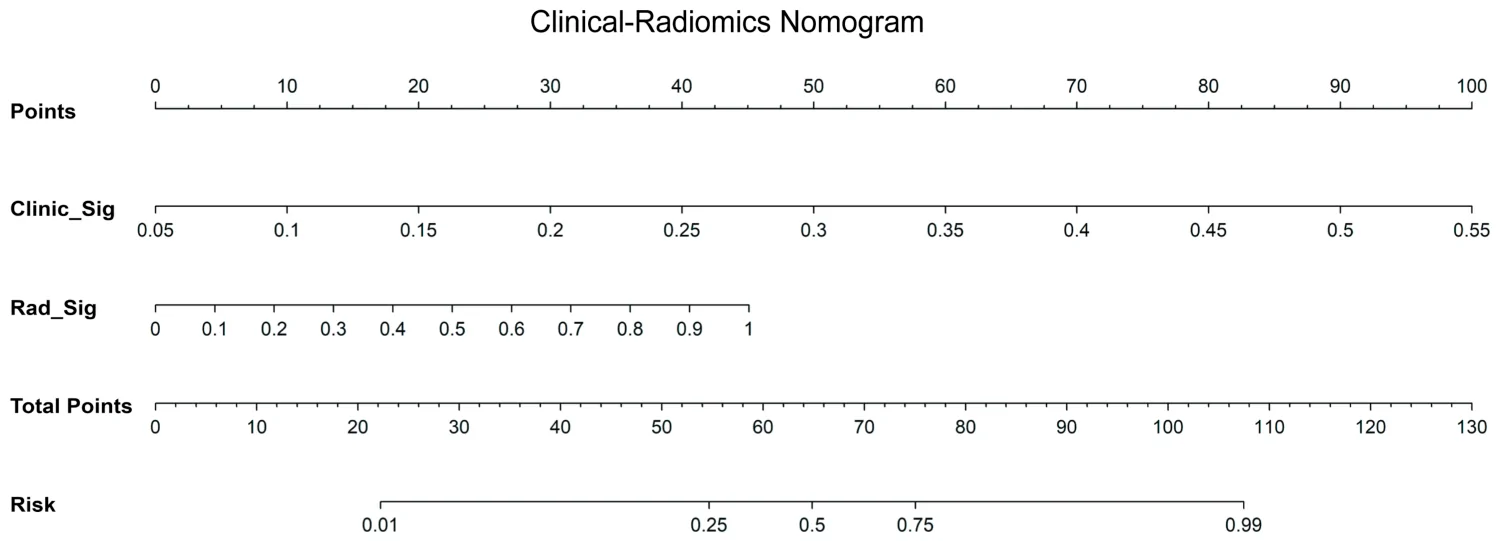
Nomogram for predicting lymphoma versus cancer of unknown primary in patients with poorly differentiated cervical cancer.

**Figure 6:**
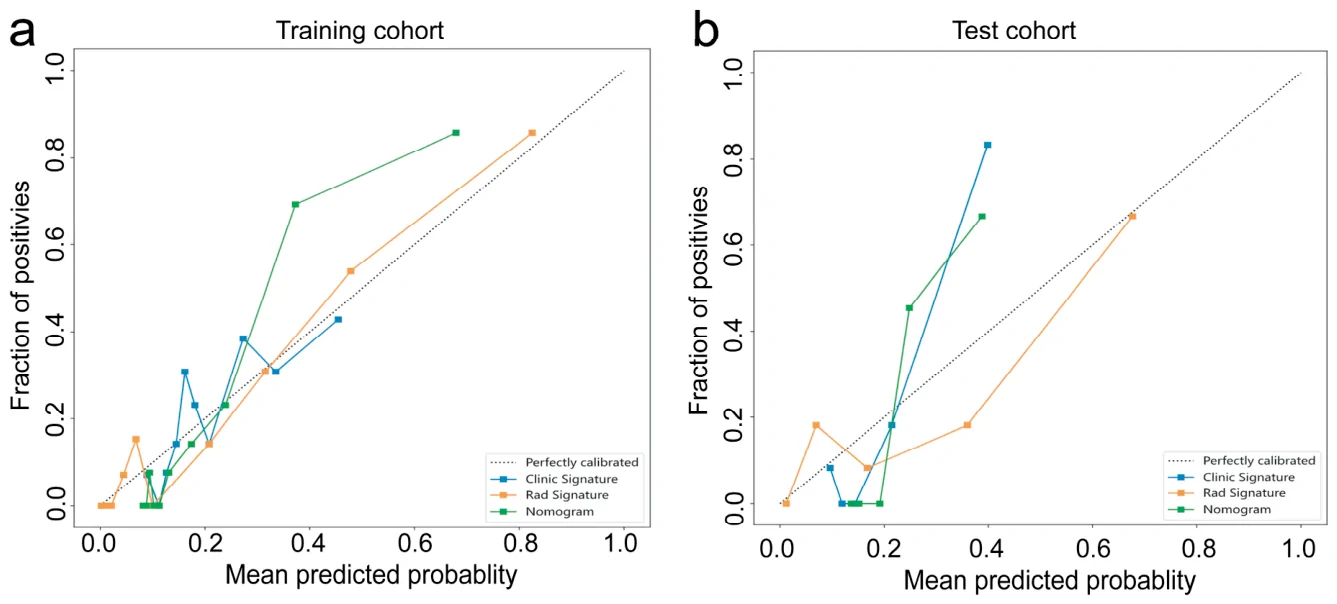
Calibration curves of the clinical–radiomics nomogram in the (**a**) training cohort and (**b**) test cohort.

**Figure 7:**
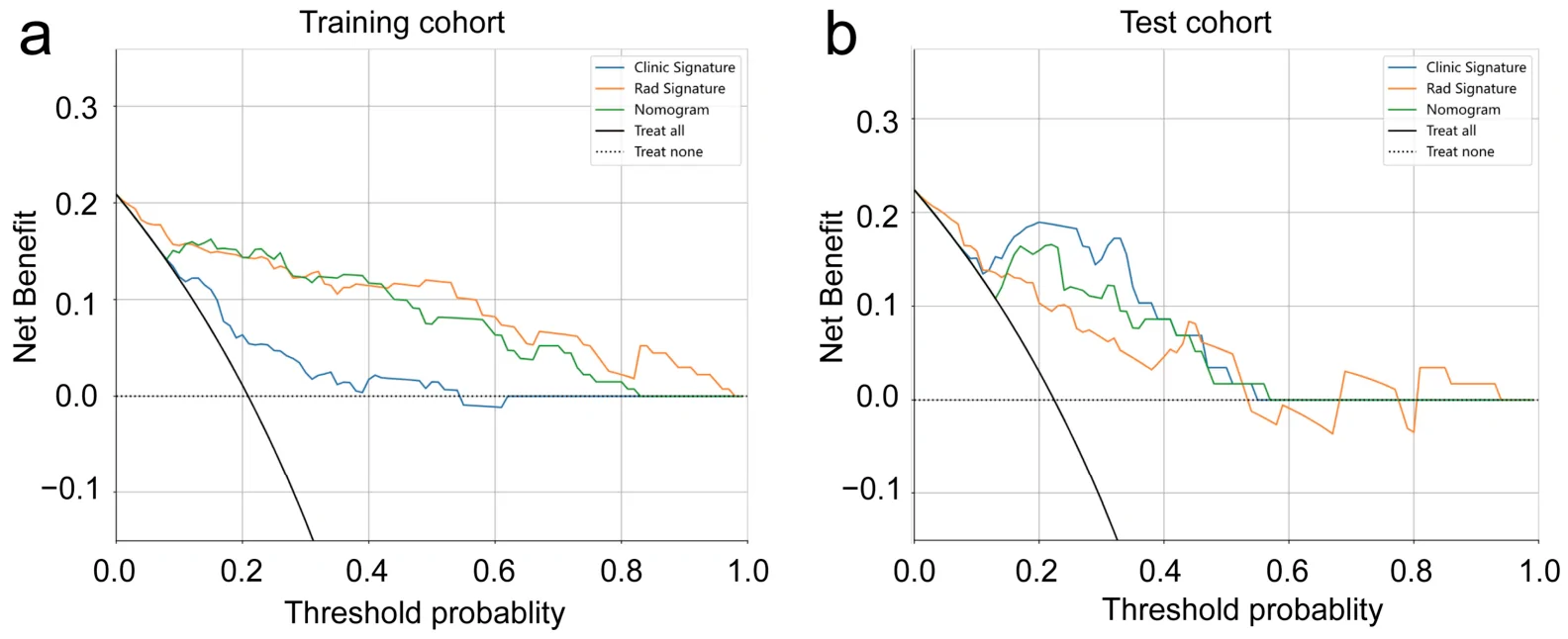
Decision curve analysis of the clinical–radiomics nomogram in the (**a**) training cohort and (**b**) test cohort.

**Table 1:** Baseline characteristics of patients in training and test cohorts.

Characteristics	Training Group (*n* = 134)			Test Group (*n* = 58)		
	HNCUP	Lymphoma	*p*-Value	HNCUP	Lymphoma	*p*-Value
Age, year	59 (22–85)	60 (33–83)	0.941	60 (29–78)	56 (26–76)	0.933
Gender						
Female	16	14	<0.001	11	8	0.022
Male	90	14		34	5	
BMI, kg/m^2^	23.2 (16.3–32.0)	23.9 (18.6–29.1)	0.282	22.3 (16.3–31.5)	22.9 (19.1–27.9)	0.305
Short diameter, cm	2.0 (0.6–5.9)	2.4 (0.9–6.8)	0.158	2.1 (0.7–5.6)	2.1 (0.9–3.9)	0.912
SUV_max_	11.6 (3.1–33.8)	19.4 (7.3–54.8)	<0.001	11.1 (3.7–27.7)	18.38 (5.7–33.1)	0.004
SUV_mean_	5.3 (2.7–11.0)	7.3 (3.6–18.6)	<0.001	5.3 (3.1–9.5)	6.9 (3.4–10.3)	0.033
MTV, cm^3^	26.5 (1.2–661.4)	46.4 (2.5–301.6)	0.198	23.1 (0.7–281.8)	27.2 (4.6–362.1)	0.229
TLG	147.2 (3.2–4411.7)	312.5 (8.8–5610.5)	0.02	136.0 (2.1–1135.8)	212.7 (15.2–2700.9)	0.041
HI	2.3 (1.1–4.0)	2.6 (2.0–4.7)	0.001	2.2 (1.2–5.5)	2.7 (1.7–4.0)	0.006

**Table 2:** Multivariate logistic regression analysis of predictors for distinguishing lymphoma from cancer of unknown primary.

Variable	OR (95%Cl)	*p*-Value
Gender	8.793 (2.801 to 27.607)	<0.001
SUV_max_	1.214 (0.269 to 5.472)	0.801
SUV_mean_	3.396 (0.786 to 14.671)	0.102
MTV	2.444 (0.650 to 9.198)	0.186
TLG	1.078 (0.294 to 3.952)	0.910
HI > 2.37	5.762 (1.422 to 23.348)	0.014

**Table 3:** Performance of the LR-based radiomics model in training and test cohorts.

Radiomics Model		AUC (95% CI)	Accuracy	Sensitivity	Specificity	PPV	NPV	F1 Score
CT	Training	0.791 (0.699–0.883)	0.687	0.857	0.642	0.387	0.944	0.533
	Test	0.817 (0.703–0.932)	0.724	0.846	0.689	0.440	0.939	0.579
PET	Training	0.828 (0.738–0.918)	0.866	0.571	0.943	0.727	0.893	0.640
	Test	0.692 (0.521–0.863)	0.707	0.538	0.756	0.389	0.850	0.452
PET/CT	Training	0.916 (0.855–0.976)	0.828	0.857	0.821	0.558	0.956	0.676
	Test	0.812 (0.682–0.942)	0.828	0.538	0.911	0.636	0.872	0.583

**Table 4:** Performance of the clinical nomogram, radiomics nomogram, and clinical–radiomics nomogram in training and test cohorts.

Model		AUC (95% CI)	Accuracy	Sensitivity	Specificity	PPV	NPV	F1 Score
Clinical nomogram	Training	0.723 (0.624–0.823)	0.552	0.857	0.472	0.300	0.926	0.444
	Test	0.923 (0.799–1.000)	0.897	0.846	0.911	0.733	0.954	0.786
Radiomics nomogram	Training	0.916 (0.856–0.976)	0.828	0.857	0.821	0.558	0.956	0.676
	Test	0.812 (0.682–0.942)	0.828	0.538	0.911	0.636	0.872	0.583
Clinical–radiomics nomogram	Training	0.924 (0.868–0.980)	0.881	0.821	0.896	0.676	0.950	0.742
	Test	0.912 (0.837–0.987)	0.810	0.923	0.778	0.545	0.972	0.686

## Data Availability

The data and code used to support the findings of this study are available from the corresponding author on request.

## Supplementary Materials

The following supporting information can be downloaded at https://www.mdpi.com/article/10.3390/bioengineering13101127/s1, Figure S1: Example segmentation image. PET image with VOI overlay in axial (a), coronal (b), and sagittal (c) views. Table S1: LASSO-selected radiomic features and their corresponding coefficients from PET and CT feature sets. Table S2: Comparative analysis of the diagnostic performance of CT feature set radiomics models using various machine learning algorithms. Table S3: Comparative analysis of diagnostic performance for PET feature-set radiomics models across various machine learning algorithms. Table S4: Comparative analysis of diagnostic performance for PET/CT feature-set radiomics models across various machine learning algorithms. Table S5: Performance comparison of the nomogram based on SUV_bw_ and SUL from paired sensitivity analysis. Table S6: Subgroup performance of the proposed nomogram stratified by BMI tertiles. Table S7: Comparison of baseline clinical characteristics and PET metabolic parameters between patients scanned on Biograph 16 HR and Biograph mCT Flow scanners. Table S8: Sensitivity analysis of wavelet basis selection for PET radiomic features.

## Abbreviations

The following abbreviations are used in this manuscript:

^18^F-FDG	^18^F-fluorodeoxyglucose
95% CI	95% confidence interval
AUC	Area under the receiver operating characteristic curve
BMI	Body mass index
coif1	Coiflet wavelet of order 1
CUP	Cancer of unknown primary
DCA	Decision curve analysis
DLBCL	Diffuse large B-cell lymphoma
EBV	Epstein–Barr virus
FUSCC	Fudan University Shanghai Cancer Center
HI	Heterogeneity index
HL	Hosmer–Lemeshow
HNCUP	Head and neck cancer of unknown primary
HPV	Human papilloma virus
HU	Hounsfield unit
ICC	Intra-class correlation coefficient
LASSO	Least absolute shrinkage and selection operator
LDH	Lactate dehydrogenase
LR	Logistic regression
MTV	Metabolic tumor volume
NPV	Negative predictive value
OR	Odds ratio
OSEM	Ordered-subset expectation maximization
PET/CT	Positron emission tomography/computed tomography
PPV	Positive predictive value
ROC	Receiver operating characteristic curve
sIL-2R	Soluble interleukin-2 receptor
SUV	Standard uptake value
SUL	Lean-body-mass-normalized SUV
SUV_bw_	Body-weight-normalized SUV
SUV_max_	Maximum standardized uptake value
SUV_mean_	Mean standardized uptake value
SWT	Stationary wavelet transform
TLG	Total lesion glucose
VOI	Volume of interest
