# Supplementary material for: Clinical–Radiomics Nomogram Based on 18F-FDG PET/CT for Distinguishing Lymphoma from Cancer of Unknown Primary in Head and Neck Metastatic Poorly Differentiated Cancer
Source: Bioengineering (Basel). 2026 Sep 27;13(10):1127. doi: 10.3390/bioengineering13101127 (PMC13701412; doi:10.3390/bioengineering13101127)
Supplement: Supplementary file 1 [file bioengineering-13-01127-s001.zip › bioengineering-4530104-supplementary.pdf]

bioengineering

MDPI

Clinical-Radiomics Nomogram Based on ^{18}F-FDG PET/CT for Distinguishing Lymphoma from Cancer of Unknown Primary in Head and Neck Metastatic Poorly Differentiated Cancer

Supplementary Materials

Supplementary Figure S1. Example segmentation image. PET image with VOI overlay in axial (a), coronal (b), and sagittal (c) views.

Supplementary Table S1. LASSO-selected radiomic features and their corresponding coefficients from PET and CT feature sets.

Supplementary Table S2. Comparative analysis of the diagnostic performance of CT feature set radiomics models using various machine learning algorithms.

Supplementary Table S3. Comparative analysis of diagnostic performance for PET feature-set radiomics models across various machine learning algorithms.

Supplementary Table S4. Comparative analysis of diagnostic performance for PET/CT feature-set radiomics models across various machine learning algorithms.

Supplementary Table S5. Performance comparison of the nomogram based on SUV_{bw} and SUL from paired sensitivity analysis.

Supplementary Table S6. Subgroup performance of the proposed nomogram stratified by BMI tertiles.

Supplementary Table S7. Comparison of baseline clinical characteristics and PET metabolic parameters between patients scanned on Biograph 16 HR and Biograph mCT Flow scanners.

Supplementary Table S8. Sensitivity analysis of wavelet basis selection for PET radiomic features.

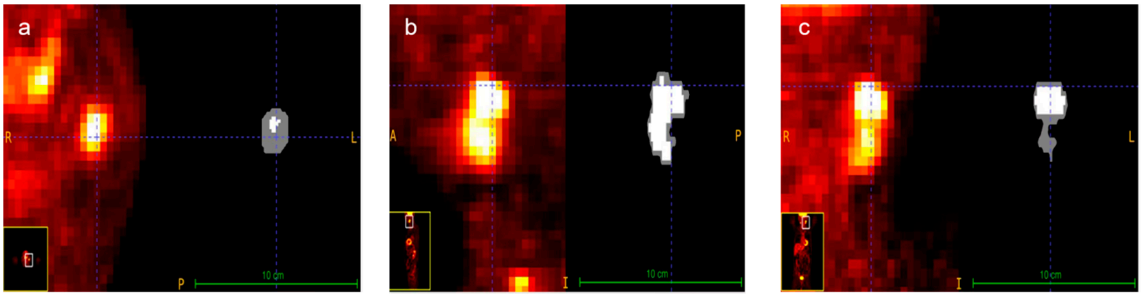

Supplementary Figure S1. Example segmentation image. PET image with VOI overlay in axial (a), coronal (b), and sagittal (c) views.

Supplementary Table S1. LASSO-selected radiomic features and their corresponding coefficients from PET and CT feature sets.

Feature names		Coefficients
Selected CT features	original_shape_Flatness	-0.041
	original_glcm_InverseVariance	0.025
	lbp-2D_firstorder_Median	-0.053
	wavelet-LHH_firstorder_Minimum	-0.015
	wavelet-LHH_glcm_Imc2	0.017
	wavelet-HLL_firstorder_Kurtosis	0.021
	wavelet-HLH_glszm_LowGrayLevelZoneEmphasis	-0.042
	wavelet-HLH_glszm_SmallAreaLowGrayLevelEmphasis	-0.022
	wavelet-HHL_glrlm_RunLengthNonUniformityNormalized	-0.028
	log-sigma-3-mm-3D_gldm_DependenceVariance	0.038
Selected PET features	log-sigma-3-mm-3D_glrlm_LongRunHighGrayLevelEmphasis	-0.002
	lbp-3D-k_glszm_ZoneEntropy	-0.022
	wavelet-LLH_glszm_GrayLevelNonUniformityNormalized	-0.007
	wavelet-LLH_glszm_SmallAreaLowGrayLevelEmphasis	-0.002
	wavelet-LHH_glszm_SizeZoneNonUniformityNormalized	0.054
	wavelet-HLL_glcm_ClusterProminence	0.008
	wavelet-HLL_glcm_Imc1	-0.022
	wavelet-HLL_ngtdm_Complexity	-0.022
	wavelet-HLH_glszm_LowGrayLevelZoneEmphasis	0.029
	wavelet-HHH_glszm_ZoneEntropy	-0.020
	exponential_glszm_SizeZoneNonUniformity	-0.013

Supplementary Table S2. Comparative analysis of the diagnostic performance of CT feature set radiomics models using various machine learning algorithms.

Radiomics models		AUC (95% CI)	Accuracy	Sensitivity	Specificity	PPV	NPV	F1 score
LR	Training	0.791 (0.699-0.883)	0.687	0.857	0.642	0.387	0.944	0.533
	Test	0.817 (0.703-0.932)	0.724	0.846	0.689	0.440	0.939	0.579
SVM	Training	0.868 (0.767-0.968)	0.903	0.786	0.934	0.759	0.943	0.772
	Test	0.703 (0.513-0.892)	0.707	0.615	0.733	0.400	0.868	0.485
KNN	Training	0.789 (0.712-0.865)	0.799	0.143	0.972	0.571	0.811	0.229
	Test	0.682 (0.521-0.843)	0.759	0.077	0.956	0.333	0.782	0.125
MLP	Training	0.800 (0.711-0.889)	0.701	0.821	0.670	0.397	0.934	0.535
	Test	0.773 (0.639-0.907)	0.638	0.846	0.578	0.367	0.929	0.512
LightGBM	Training	0.868 (0.805-0.931)	0.799	0.857	0.783	0.511	0.954	0.640
	Test	0.650 (0.462-0.839)	0.793	0.308	0.933	0.571	0.824	0.400
Random Forest	Training	0.999 (0.998-1.000)	0.970	0.857	1.000	1.000	0.964	0.923
	Test	0.752 (0.580-0.924)	0.759	0.615	0.800	0.471	0.878	0.533
XGBoost	Training	0.998 (0.995-1.000)	0.985	0.929	1.000	1.000	0.981	0.963
	Test	0.718 (0.542-0.894)	0.759	0.538	0.822	0.467	0.860	0.500
ExtraTrees	Training	1.000 (1.000-1.000)	0.791	0.000	1.000	0.000	0.791	-
	Test	0.709 (0.541-0.878)	0.810	0.308	0.956	0.667	0.827	0.421

LR,Logistic Regression; KNN, K-Nearest Neighbors; SVM, Support Vector Machine; MLP, Multi-Layer Perceptron; Light GBM, Light Gradient Boosting Machine; XGBoost, Extreme Gradient Boosting; ExtraTrees, and Extra Trees; AUC, area under curve; CI, 95% confidence interval; PPV, positive predictive value; NPV, negative predictive value. The overall performance across models was moderate, with significant overfitting observed in several ensemble methods. No single model demonstrated consistently high and generalizable performance, suggesting CT features alone may provide limited discriminative power for this task.

Supplementary Table S3. Comparative analysis of diagnostic performance for PET feature-set radiomics models across various machine learning algorithms.

Radiomics models		AUC (95% CI)	Accuracy	Sensitivity	Specificity	PPV	NPV	F1 score
LR	Training	0.828 (0.738-0.918)	0.866	0.571	0.943	0.727	0.893	0.640
	Test	0.692 (0.521-0.863)	0.707	0.538	0.756	0.389	0.850	0.452
SVM	Training	0.873 (0.845-0.988)	0.873	0.893	0.868	0.641	0.968	0.746
	Test	0.690 (0.521-0.863)	0.690	0.615	0.711	0.381	0.865	0.471
KNN	Training	0.879 (0.822-0.936)	0.843	0.429	0.953	0.706	0.863	0.533
	Test	0.579 (0.401-0.758)	0.793	0.077	1.000	1.000	0.789	0.143
MLP	Training	0.828 (0.741-0.915)	0.821	0.643	0.868	0.562	0.902	0.600
	Test	0.721 (0.572-0.871)	0.655	0.846	0.600	0.379	0.931	0.524
LightGBM	Training	0.924 (0.879-0.968)	0.821	0.929	0.792	0.542	0.977	0.684
	Test	0.680 (0.502-0.859)	0.759	0.615	0.800	0.471	0.878	0.533
Random Forest	Training	0.998 (0.996-1.000)	0.970	0.964	0.972	0.900	0.990	0.931
	Test	0.713 (0.573-0.852)	0.690	0.462	0.756	0.353	0.829	0.400
XGBoost	Training	1.000 (1.000-1.000)	0.993	0.964	1.000	1.000	0.991	0.982
	Test	0.694 (0.520-0.868)	0.672	0.615	0.689	0.364	0.861	0.457
ExtraTrees	Training	1.000 (1.000-1.000)	0.791	0.000	1.000	0.000	0.791	-
	Test	0.642 (0.448-0.835)	0.724	0.462	0.800	0.400	0.837	0.429

LR,Logistic Regression; KNN, K-Nearest Neighbors; SVM, Support Vector Machine; MLP, Multi-Layer Perceptron; Light GBM, Light Gradient Boosting Machine; XGBoost, Extreme Gradient Boosting; ExtraTrees, and Extra Trees; AUC, area under curve; CI, 95% confidence interval; PPV, positive predictive value; NPV, negative predictive value. While the LR model showed relatively stable generalization, other models exhibited high variance between training and test sets or severe overfitting. The performance of PET-based models was not consistently superior, indicating that metabolic features alone might be insufficient for optimal prediction.

Supplementary Table S4. Comparative analysis of diagnostic performance for PET/CT feature-set radiomics models across various machine learning algorithms.

Radiomics models		AUC (95% CI)	Accuracy	Sensitivity	Specificity	PPV	NPV	F1 score
LR	Training	0.916 (0.855-0.976)	0.828	0.857	0.821	0.558	0.956	0.676
	Test	0.812 (0.682-0.942)	0.828	0.538	0.911	0.636	0.872	0.583
SVM	Training	0.973 (0.933-1.000)	0.940	0.929	0.943	0.812	0.980	0.867
	Test	0.785 (0.619-0.950)	0.793	0.692	0.822	0.529	0.902	0.600
KNN	Training	0.851 (0.784-0.918)	0.851	0.357	0.981	0.833	0.852	0.500
	Test	0.736 (0.590-0.881)	0.741	0.154	0.911	0.333	0.788	0.211
MLP	Training	0.915 (0.850-0.981)	0.866	0.857	0.868	0.632	0.958	0.727
	Test	0.827 (0.710-0.945)	0.759	0.769	0.756	0.476	0.919	0.588
LightGBM	Training	0.946 (0.901-0.990)	0.858	0.893	0.849	0.610	0.968	0.725
	Test	0.553 (0.365-0.741)	0.776	0.154	0.956	0.500	0.796	0.235
Random Forest	Training	1.000 (1.000-1.000)	0.985	0.929	1.000	1.000	0.981	0.963
	Test	0.649 (0.483-0.814)	0.793	0.231	0.956	0.600	0.811	0.333
XGBoost	Training	0.993 (1.000-1.000)	0.993	0.964	1.000	1.000	0.991	0.982
	Test	0.517 (0.494-0.836)	0.517	0.769	0.444	0.286	0.870	0.417
ExtraTrees	Training	1.000 (1.000-1.000)	0.791	0.000	1.000	0.000	0.791	-
	Test	0.750 (0.578-0.922)	0.810	0.308	0.956	0.667	0.827	0.421

LR,Logistic Regression; KNN, K-Nearest Neighbors; SVM, Support Vector Machine; MLP, Multi-Layer Perceptron; Light GBM, Light Gradient Boosting Machine; XGBoost, Extreme Gradient Boosting; ExtraTrees, and Extra Trees; AUC, area under curve; CI, 95% confidence interval; PPV, positive predictive value; NPV, negative predictive value. Based on comparative analysis, the LR model trained on the fused PET/CT feature set showed the most balanced and generalizable performance among all evaluated algorithms (Test AUC: 0.812, Sensitivity: 0.538, Specificity: 0.911). Although more complex models like SVM and Random Forest achieved near - perfect performance on the training set, they overfitted significantly, as shown by large drops in AUC and sensitivity on the independent test set. The selected LR model had robust specificity and maintained

a clinically informative AUC in validation. Moreover, its simplicity, interpretability, and stable generalization across cohorts make it a more suitable and reliable option for clinical translation than the overfitted alternatives. This model also provides the optimal radiomics signature for integration with clinical parameters in the subsequent nomogram construction.

Supplementary Table S5. Performance comparison of the nomogram based on SUV_{bw} and SUL from paired sensitivity analysis.

Metric	SUV_{bw} (paired re-analysis)	SUL (paired re-analysis)	Δ
Nomogram train AUC	0.924 (primary; n=134)	0.914 (sensitivity; n=133)	–0.010
Nomogram test AUC	0.912 (primary; n=58)	0.877 (sensitivity; n=58)	–0.035
Rad_score Spearman r	—	0.86 (train) / 0.92 (test)	—

Supplementary Table S6. Subgroup performance of the proposed nomogram stratified by BMI tertiles.

Cohort	BMI strata (kg/m^2)	Total	AUC (95% CI)
Training	Low (<22.1)	45	0.924 (0.869-0.979)
	Middle (22.1-25.0)	43	0.918 (0.84-0.98)
	High (>25.0)	46	0.928 (0.87-0.98)
	Overall	134	0.924 (0.868-0.980
Test	Low (<22.1)	26	0.910 (0.80-0.98)
	Middle (22.1-25.0)	24	0.915 (0.82-0.98)
	High (>25.0)	8	0.890 (0.70-0.99)
Test	Overall	58	0.912 (0.837-0.987)

Note: Test-set subgroups suffered from very small positive event counts and should be interpreted cautiously. Spearman correlation between BMI and radiomics score: $r = 0.062$, $p > .05$.

Supplementary Table S7. Comparison of baseline clinical characteristics and PET metabolic parameters between patients scanned on Biograph 16 HR and Biograph mCT Flow scanners.

**Variable**	**Biograph 16 HR (n = 128)**	**Biograph mCT Flow (n = 64)**	**P-value**
Clinical characteristics			
Age, years, median (IQR)	59 (52-65)	60 (49-66)	0.899
Male sex, n (%)	98 (76.6%)	45 (70.3%)	0.447
BMI, kg/m², median (IQR)	23.1 (21.2-25.9)	23.0 (20.8-24.5)	0.291
PET quantitative parameters			
SUVmax, median (IQR)	13.8(10.1-19.0)	11.4 (8.3-17.1)	0.071
SUVmean, median (IQR)	5.5 (4.6-7.0)	5.3 (4.3-6.6)	0.270
Heterogeneity index (HI), median (IQR)	2.4 (2.0-2.7)	2.2 (1.8-2.6)	0.063

Note: Continuous variables were compared using the Mann-Whitney U test; categorical variables were compared using the chi-square test. All P-values > 0.05 indicated no significant inter-scanner differences in baseline patient and lesion metabolic features.

Supplementary Table S8. Sensitivity analysis of wavelet basis selection for PET radiomic features.

Wavelet basis	Training AUC	Training 95% CI	Test AUC	Test 95% CI
coif1 (original)	0.924	0.868-0.980	0.912	0.837-0.987
db2	0.921	0.864-0.978	0.905	0.829-0.981
sym3	0.920	0.862-0.978	0.902	0.825-0.979
bior2.2	0.918	0.860-0.976	0.898	0.821-0.975
